# Waiting for markets to change me—High-stakeholders' views of antibiotic use and antibiotic resistance in pig production in Brazil

**DOI:** 10.3389/fvets.2022.980546

**Published:** 2022-09-16

**Authors:** Rita Albernaz-Gonçalves, Gabriela Olmos Antillón, Maria José Hötzel

**Affiliations:** ^1^Laboratório de Etologia Aplicada e Bem-Estar Animal, Departamento de Zootecnia e Desenvolvimento Rural, Universidade Federal de Santa Catarina, Florianópolis, SC, Brazil; ^2^Instituto Federal Catarinense, Campus Santa Rosa do Sul, Santa Rosa do Sul, SC, Brazil; ^3^Veterinary Epidemiology Unit, Department of Clinical Sciences, Swedish University of Agricultural Sciences, Uppsala, Sweden

**Keywords:** AMU, AMR, animal welfare, professionals, swine, polices, One Health

## Abstract

Overuse of veterinary antibiotics is a risk factor for antimicrobial resistance (AMR), which is a global public health emergency. More than 70% of the antibiotics consumed worldwide are used in farm animals, mainly in poultry and pig herds. Brazil is the fourth largest pork producer globally and the second-largest user of antibiotics in animals. Qualitative research can help understand the complexities around antibiotic use (AMU) in Brazilian pig herds and identify stakeholders' attitudes concerning the rational AMU and AMR in the production chain. This study aimed to explore the knowledge and attitudes of high-level professionals in the animal production chain about AMU and AMR in pig farming, the relationship with pig welfare and AMU in Brazil. We conducted 32 in-depth interviews with individuals active in the pig industry. The majority of the participants considered AMU excessive and inappropriate in pig farms in Brazil. However, attitudes toward a restrictive AMU scenario in Brazilian pig farms were predominantly negative, justified by economic, sanitary and social barriers. These included unsatisfactory management and biosecurity conditions in pig farms that, in their opinion, justify AMU to prevent diseases; issues surrounding prescription and acquisition of veterinary drugs; and employment and income relationships arising from the sale of antibiotics. The views of high-level professionals in the Brazilian livestock chain reveal antibiotics as a structural element that enables pig production. Antibiotics were viewed as essential resources for producing cheap food. Foreign markets were considered the most relevant driver of change in AMU practices rather than pressure from Brazilian consumers. A common belief expressed was that AMR is more associated with the inappropriate AMU in human medicine than in the livestock sector. Resistance to change in these stakeholders may hinder the implementation of future public policies to restrict the use of antibiotics in Brazil. Our findings suggest that successful measures to deal with the AMU/AMR challenges in the pig chain shall not be rooted in personal behavior change. Instead, honest interdisciplinary dialogues and structural changes are needed to define common grounds and a way forward to break the cycle perpetuating antibiotics as structural commodities.

## Introduction

Some authors understand antimicrobial resistance (AMR) as an imminent “tragedy of the commons” (1), of anthropogenic nature and analogous to climate change in terms of its challenges (1–3). Although the resistance process is a natural defense mechanism of bacteria, it can be intensified by the misuse of antibiotics both in humans and animals. Inappropriate use of antibiotics (AMU) in human health is a powerful inducer of AMR. However, about 73% of the volume of antibiotics produced in the world is used in intensive livestock production (4), mainly in sub-therapeutic doses to promote weight gain or prevent diseases in herds (5). This potentiates the spread of resistant bacteria, especially in the environment (6, 7). AMR transmission can occur *via* contaminated food and *via* the environment (7, 8), as well as through direct contact with contaminated animals, which puts farmers and slaughterhouse workers especially at risk (8–10). Resistant bacteria genes have been identified in healthy pigs, in pig manure, pen floor, surface soil, sewage, urban water reservoirs, and food (11–15). Many studies in several countries bring to light the misuse of antibiotics in pig farming, for example in Brazil (16), Belgium (17, 18), Vietnam (19), Cambodia (20), and Italy (21). In part this is related to stressful conditions of intensive pig farming, as stressed pigs are immunocompromised, increasing their susceptibility to disease (22, 23). In these conditions, a common strategy to prevent diseases is the use of antibiotics, often in place of preventive practices (16, 24). Altogether, this suggests that a more rational AMU in farm animals may help decrease AMR (25).

In response to the threat of bacterial resistance to antibiotics, public policies for its prudent use have been discussed and implemented in several countries (26–28). Brazil is the 4th largest pig producer in the world, with a large and growing domestic market, and where the export of pork is an important activity for the agribusiness sector (29). The public health emergency involving AMR adds pressure on countries that export agricultural products, including pig meat, to adapt to international requirements for AMU in the future (30, 31). The Brazilian Ministry of Health, together with the Ministry of Agriculture and other entities, developed a program to start discussions about policies on the use of antibiotics in Brazil, the PAN-BR (32). The success of such a programme will depend on the ability to work with a bottom-up approach that includes practical and situated transdisciplinary knowledge of AMU in the pig production chain (33). Although pig farmers are the stakeholders of the pig production chain administering the drugs, the production chain comprises different social actors whose views and decision-making significantly impact Brazil's paths concerning the international call to curb AMR. Yet, little is known of the knowledge and views of these other stakeholders (here referred as high-level professionals) regarding the country's AMU/AMR challenges. Qualitative research can help capture elements to understand the social context in which AMU and AMR are involved in pig farming.

Exploring the attitudes of stakeholders in the pig production chain can identify knowledge gaps and barriers to implementing public policies and help formulate strategies that may encourage rational AMU (34). The objective of this study was to explore the knowledge and attitudes of high-level professionals in the animal production chain about AMU in pig farming, AMR, animal welfare and measures for prudent AMU in Brazil.

## Materials and methods

This study is part of the research project “Knowledge and attitudes of Santa Catarina's pig farming on antibiotics, bacterial resistance and animal welfare” carried out by the Laboratory of Applied Ethology at the Federal University of Santa Catarina—LETA-UFSC. This study followed a qualitative approach to obtain a detailed account of pig production stakeholders' (other than farmers) views on antibiotics and animal welfare, acquired through in-depth semi-structured interviews. The concept of AMR covers a variety of drugs. However, the focus of this study is antibiotics, due to the amount of use of these drugs and their importance in the pig production chain. This study was approved by the Human Research Ethics Committee of the Federal University of Santa Catarina (CEPSH / UFSC) under decision n. 2.562.764.

### Participants' recruitment

The study sought to complement the knowledge obtained from pig farmers already described by this group (16). We sought professionals with a background education in veterinary and animal science, agronomy, animal nutrition or pharmacology, linked to different roles or organizations of the pig production industry. The initial participants (informants) were recruited from personal contacts of RAG, who is a qualified veterinarian by the Federal University of Pelotas, Brazil, with practical experience in pig farms, currently a permanent faculty at the Instituto Federal Catarinense. From these initial conversations, subsequent names, roles and organizations of interest to our query were identified and interviewed. These included persons with an affiliation to official Brazilian entities or bodies.

Participants were interviewed individually between April 2018 and July 2019 by RAG, in Brazilian Portuguese. According to the participants' availability, interviews were done either in person, *via* telephone or videoconference. The participant could speak freely about the topics covered in the interviews, which lasted between 30 min and 2 h. In accordance with the CEPSE-UFSC regulations and the current legislation (Resolution 466/2012) for research work with humans, the participants received and signed a Term of Free Consent and Clarification (TFCC) before the interviews started. This term contained all the information about the research, identifying the persons responsible for the project and giving detailed information regarding the participants' rights. The TFCCs were sent by e-mail or delivered during the in-person interviews.

The participants were 32 individuals (69% men and 31% women) with 3–30 years of professional activity, of which 69% were veterinarians, 22% animal scientists, and 9% held other professions (agriculture technicians or biologists). The group belonged to different sectors of the pig production chain and to different hierarchies within each sector. It included veterinarians from government animal health agencies, agricultural technicians and veterinarian public extensionists and private advisors from associations and agribusiness, sales representatives from the pharmaceutical sector, animal nutritionists, official agricultural inspectors, researchers from federal and state research institutions and faculty at veterinary schools. Information on AMU in pig farming was gathered from participants that were directly linked to the pig production chain. Some participants represented important entities and associations in the pig production chain, where they held coordination functions. To ensure the anonymity of the participants we numbered them sequentially from 1 to 32 and did not specify their profession or role within the pig production chain. The characterization of the professionals and their relationships in the production chain are described in item “Social Pillar” of the results section.

### Interview script

In interviews we discussed the following central issues with the participants; antibiotics importance, AMU in Brazilian pig farming, rational AMU, AMU polices, AMR, relationship between AMU and AMR (animals and humans), animal welfare, relationship between AMU and animal welfare and Brazilian consumers (see script in Supplementary Table 1). We asked participants some questions to help characterize the intensive pig breeding system in Brazil, understand the production chain, and define issues previously identified in interviews with farmers (16, 35). At the end of the interviews, we presented the participants with a hypothetical scenario of restricted AMU in line with international models of rational AMU. Participants were asked to give their opinion on the feasibility of this scenario within the Brazilian pig production context and to point out measures they judged necessary to adapt the production chain to such scenario.

### Data analysis

Closed responses were organized with the help of Microsoft Excel and summarized using descriptive analysis. Qualitative data were analyzed with the aid of the NVivo Qualitative Data Management Program (version 11, 2015; QSR International Pty Ltd., Doncaster, VIC, Australia) using a thematic analysis approach [see (36)] to provide a rich and detailed, yet complex account of data. All interviews were transcribed in Portuguese, verbatim by RAG. The transcribed text was read several times thoroughly by RAG and MJH and coded in central themes arising from the data. Codes were initially identified by RAG to capture the salient features of the dataset. Through interactive discussions, codes were detailed and developed into themes by the three authors. Representative quotes of themes were selected for final discussion and presentation and translated to English by MJH. The participants' quotes are described in Table 1. For example, P17.a refers to the first excerpt from the interview with Participant 17; P3b is the second excerpt quoted from the interview with Participant 3.

**Table 1:** Quotes by high stakeholders.

**Importance of antibiotics in the production chain**
P17a “In the model developed in Brazil for the production of pigs they (antibiotics) are essential. Today it is not possible to produce properly with high productivity without the participation of antibiotics, because it is based on large-scale production, with high technology”. P1a “Antibiotics are essential and necessary for health control and treatment of clinical conditions”.
**Production chain–Social Pillar** P31a “In the company we were 3 veterinarians. We met twice a year; we defined the so-called winter and summer programs. We met in April and October and, with presence of a consultant, made field visits, collected material. And within the challenges that were found in the field, mainly enteric and respiratory issues, we defined the use of medicated feed for all animals”.
P30a “The company's agricultural technician goes to the farm and leaves one or two bottles of a certain antibiotic. For diarrhea, the farmer knows that he can only use that product. He has a list of what antibiotics are and what the deficiency is, what it is for. The pig farmer has technical guidance for this approach”.
P26a “If there is a health problem in the group, the producer calls the technician and he goes there to take a look at it. If there are any more serious health problems, the veterinarian follows up and, from there, he decides whether to medicate or not”.
**Production chain–Sanitary Pillar** P26b “Many farms that do not have any isolation, have no green barrier, no disinfection arch, no fence, bathing for you to enter and leave the farm, control of the entry and exit of people”. P4b “When it comes to hygiene, disinfection, pest control, in Brazil you do not enter a farm that is not full of flies, which doesn't have rats. Nowadays you can't say that we do pest control or hygiene in an efficient way”. P30b “If we take the antibiotics tomorrow and do not use any type of antibiotic in feed, for example, we would work with a much higher mortality rate, because today we are not prepared for that”. P20a “So I think that at first, we will suffer major problems with diarrhea and respiratory diseases. This also happened in other countries and, if you look at it, maybe we will see an increase in the use of antibiotics. Not preventive, but curative”. P15a “You would have to work hard to improve conditions, things we already know, biosecurity, vaccination… Some protocols for the eradication of some diseases that can be eradicated within the production system. Working with production pyramids, with the health part that we know, reducing the mixture of animals”. P15b “The preventive use of antibiotics will be more difficult to eliminate, because we have many diseases on the farms. So, we will have to work on this part of risk factors and contamination. It is complicated to deal with some diseases without the preventive medication”. P32a “In all phases, a lot of antibiotics are used. In the nursery, it is insane”
P1b “... in a situation where you need to make a prophylactic use throughout the animal's life, I think this is wrong. But at specific stages, considering the environment, due to the stress suffered by the animals, in a short period, in dosages that are not growth promoters, but appropriate to avoid a major disorder, of mortality, I understand it as acceptable”.
**AMU/AMR** P8a “It has not yet been possible to prove, the link of migration of possible resistance from the animal area to the human area (...) but as a precaution we are going to ban the use, that was it what the European Union did”.
P15c “Because although it is a global alarm, and the WHO estimates that 2050 more people will die from bacteria than from a car accident, we need to do a risk assessment to get it right (...) Because if you get the diagnosis wrong you can zero out the use of antimicrobials in agriculture without solving the problem in humans”.
**Production chain- Economic Pillar** P30c “Lately, we have been receiving guidelines to stop using antibiotics for growth promotion. There are molecules that we are no longer using, such as colistin, which before we used a lot as a growth promoter and, today, we can no longer use”. P1c “I see that science has evolved; research has evolved considerably. In the past, it was a need because of lack of alternatives. … Today there are alternatives that give the same result as the antibiotic. To insist on antibiotics as a growth promoter is to be outdated. …there are several products and several tools that can be used, such as probiotics, to favour a more balanced microbiota, a positive microbiota, so that animals can defend themselves better. But that works in a minor challenge condition, it's very difficult for you to have something that works like antibiotics in these alternatives.” P15d “In terms of money, it is a great challenge to try to replace this food conversion and economic gain that the antibiotic as a growth promoter has in a country like ours”. P6a “Producing without antibiotics costs more, so you have to have money to pay for it, because you will lose in efficiency. … we cannot sell in a low-profit market like Brazil, where there are many hungry people, without money to pay for this difference in cost”. P8b “Antibiotics are not essential, without them you will produce, you will have the production of safe food. But perhaps you will not have a competitive product in the market outside Brazil”.
P12a “In my opinion, as an animal scientist, about animal welfare, you have to have the maximum performance, productivity, produce respecting the premises of animal welfare. The fact is, how much does it cost to the production system? (...) it still pays off financially to disrespect the animal welfare system”. P9b “Pig farmers work with very low product margins and this means that they have to obtain financing for working capital, or to adapt the farms to environmental legislation, and there is little room for people to make structural changes in favour of animals”.
P8c “Part of animal welfare is that the animal is healthy. … So, if you do prophylaxis, you are generating welfare…. by giving a prophylaxis (referring to antibiotics) you will reduce animal suffering…” P14a “Better conditions for animal welfare in farming could help reduce antibiotics, but withdrawing the antibiotic could harm animal welfare”. P6b “The first requirement for sustainable production is well-being, bioclimatology, temperature, humidity, thermal stress. This is essential. If you have thermal comfort, your animal is producing. Comfort and welfare are essential. There is no discussion, you have it or you do not produce”. P5a “An animal with well-being conditions met is certainly a more productive animal, I have no doubt about it”. P9c “I will call it a lack of animal welfare. I see a direct connection between these issues. We have pigs that live in a very different environment from the one where they evolved, with significant behavioural limitations, with agonistic behaviours, chronic stress that generates low immunity. And then we have the prophylactic use of antibiotics”. P1d “Animal welfare is also related to the use of antibiotics. If I need to use antibiotics for an animal to achieve animal welfare and reach its genetic potential, the animal is not necessarily in a welfare condition.” P14b “There is the other side of the industry, which is that of medicines ... a lot of people who depend on it, who earn money from it, employment with it, selling medicine ... “ P10a “We know, both in human and animal health, there is a great influence of laboratories ... the more they are used (antibiotics), the more they sell, the more they earn money. And, of course, we (veterinarians) also profit…” P23a “People do not seek to know what the reality is. I think that there is a great lack of knowledge and some prejudices, but when it comes to consuming nobody cares. Meat is cheap in the supermarket, nobody will look there and wonder, does this have antibiotics or not. They go there, buy and eat”. P17b ”I think that in Brazil, not much yet (referring to concern with AMR). Brazilians are very concerned about eating, about consuming”. P18a “There is a growing niche of people concerned with health in general, who give a lot of importance to organic products, cleaner products, this is a fast-growing market, but in Brazil it is still emerging”. P1e “I think this is a new market, of recent years (...). As the economy stabilizes, people have more conditions and more information, people worry. The percentage of vegetarians and vegans looking for antibiotic-free products is increasing”.
**Antibiotic dependence**
P30d “In general, today's pig farming would not be prepared for a ban on the use of antibiotics. We still have pathogens that cause great losses in pigs. Today we would not be able to have this drastic restriction in a short time”. P17c “I think it is not only viable, but it will happen. If we don't take the measures for love, we will take the measures for pain. And this, I believe, will happen in Brazil. It is already happening, right? at some level. So, I think using antibiotics prudently is a way of no return for us”. P21a “If, suddenly, the foreign market does not accept the Brazilian product because it does not submit to the rule… people pay a lot of attention to what comes from outside … this will end up being a motivator for the policies to be established“. P5b “Brazilians are characterized by the flexibility and adaptability to market requirements and always comply, with the objective of maintaining the status of one of the largest producers and exporters in the world”.
**Changes and law** P28a “Having regulation, because the Brazilian is moved by regulations. Making access more difficult (...) There has to be some purchase control”. P1f “So when we do a control, unfortunately, our culture starts from this principle: if there is a regulation, a stricter punishment, then it happens. Leaving it just for common sense, for the good will of people, it is more difficult to make a significant change”. P31b “First step, to work, you need inspection” P21b “First thing is the technical training of professionals who are already in the field. I think that if we are not able to change this professional, through training, it will be difficult to have a good adherence”. P9d “I don't know if we will have the political will to implement measures to control antibiotics political system in Brazil”.

*P*, Participant. Numbers = 17.a refers to the first excerpt from the interview with Farmer 17; F3b is the second excerpt quoted from the interview with Farmer 3.

## Results

The participant's accounts pointed to antibiotics as material infrastructure of pig production, with such infrastructure consisting of three pillars: social, health, and economic. Participants trusted antibiotics and credited them for the success in the production rates and health control of pig herds. They considered these drugs as an essential part of a technological package aimed at high productivity (P17a, P1a). First, we describe the social, health, and economic pillars that are supported by antibiotics and identify where the social actors in the production chain are inserted in this context. Then, we describe the views of the participants regarding the potential consequences of AMU restriction policies for the Brazilian pig industry, which reveals a perception of dependence on antibiotics and the problems of its enforcement.

### Social pillar—The connection between the social actors in the pig production chain

**Figure 1:**
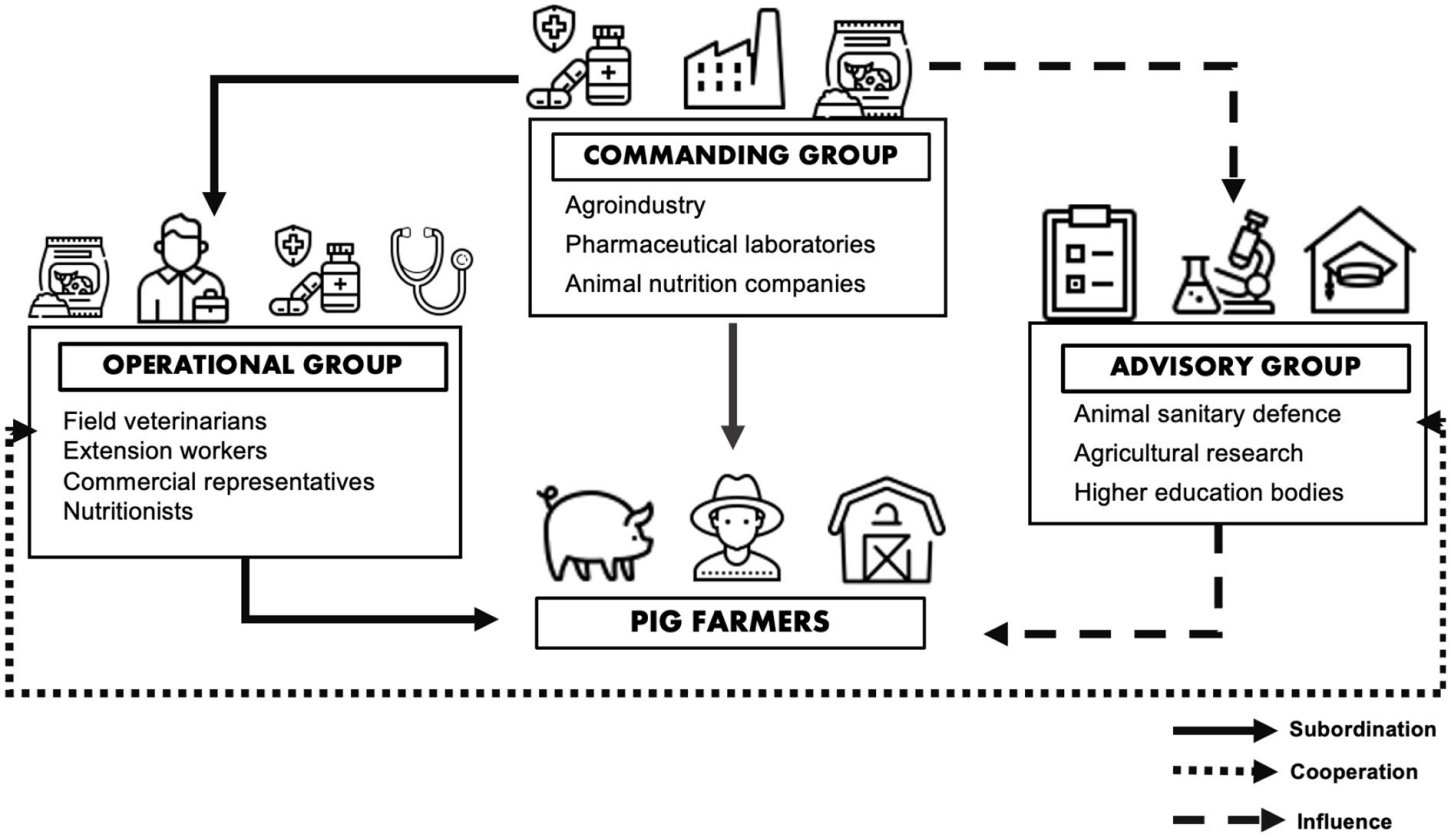
Description of the groups that make up the social pillar and their relationships.

In the social pillar, we identified the relationship of the different social actors in the pig production chain with antibiotics. Based on the participants' report, we defined the animal production chain and its social relations in three social/interest groups that we labeled as commanding, operational and advisory, respectively. Figure 1 illustrates the activities and roles of the different groups of actors and their interactions.

The **commanding group** was made up of the most stable elements in the production chain and was the one with the most significant influence on the other stakeholders. Agroindustry, the pharmaceutical laboratories, and animal nutrition companies belonged to this category and had high importance and prestige within the chain. Pharmaceutical laboratories and nutrition companies supplied antibiotics and nutritional inputs, respectively, both essential for the production and productivity of the agroindustry and employed professionals to carry out the production practices that met their demands. The commanding group determined the rules to be applied to the production system through the economic subordination of professionals and farmers and their influence on political groups that defined state and federal laws (for details, see (16). Thus, the relationship among the commanding agents was one of mutually reinforcing and preservation of the production chain. Most stakeholders from this stratum that we initially invited were unwilling to participate in the study. Thus, the information about this group arises from 3 interviews with stakeholders from the group and reports of individuals in the other two groups.

The **operational group** included professionals who carried out the rules imposed by the commanding group and included 14 of the participants in this study. It was composed of professionals with higher education or agricultural technical training who worked as employees of companies or as self-employed professionals linked to the commanding group at some level. These were field professionals (extension workers) including veterinarians, nutritionists, and other professionals, as well as commercial representatives. Field veterinarians and other professionals were part of the technical staff of the agrobusiness. It was up to the veterinarians to define the antibiotic protocols to be adopted in the herds (P31a). These protocols were defined through negotiations with commercial representatives linked to the pharmaceutical companies, involving discounts or incentives for mass purchase by agroindustry. Nutritionists worked for animal nutrition companies that provided services to agrobusiness, individual farmers, and feed factories The agrobusiness' nutritional programs were defined among nutritionists, veterinarians and agricultural technicians. Extension workers or field professionals executed the protocols defined by the veterinarians and nutritionists and supervised the practices adopted by the farmers (P30a).

The extension workers maintained direct contact with the farmers and constituted the main link between the farmers and the agrobusiness (P26a). The commercial representatives of the pharmaceutical laboratories had contact with all groups through the sale of medicines and agricultural inputs. Several veterinarians provided free technical assistance to the farmers who bought their medicines, a relationship that some viewed as a conflict of interest (P4a).

The operational agents maintained a subordinate relationship or had direct financial link with the commanding agents through employment or income from the sale of their products.

The **advisory group** was made up of animal sanitary defense professionals (linked to state or federal health agencies), agricultural research and higher education bodies; 15 of the respondents belonged to this statement of the production chain. The animal sanitary defense group developed the rules and inspected food factories, slaughterhouses, and commercial warehouses. In addition, the advisory agents participated in preparing technical normative instructions that regulate the production of food of animal and vegetable origin that supply the domestic and export markets. Agricultural researchers were related to farmers and to the three components of the commanding group (i.e., the agrobusiness, nutrition companies and pharmaceuticals), via scientific research that was of interest to these groups. Finally, higher education institutions also carried out research directly or indirectly associated with the operational groups. They were also responsible for the training and continuous education of professionals (veterinarians, nutritionists, agronomists, and agricultural technicians). Thus, higher education institutions, agricultural research institutions and the sanitary defense organizations had a formative and guiding role, offering the recommendations that lead the professionals of the operational group, as well as farmers.

### Sanitary pillar—“We need antibiotics because our environmental conditions are bad”

Participants believed that hygiene and biosecurity conditions of the farms required the use of prophylactic antibiotics (P26b, P4b). In fact, the potential restriction to the prophylactic use of antibiotics was considered a threat to the maintenance of the health of herds in the current conditions (P30b) and some argued that this could result in an increase in AMU for curative purposes (P20a). Importantly, despite being opposed to the possibility of legal restrictions on AMU, the participants were aware of the measures that would be necessary in order to adopt rational AMU strategies (P15a).

One of the main concerns expressed by the participants regarding the restriction of AMU referred to the potential difficulty in controlling the diseases of the herds. It is important to note that the participants' insecurities referred to the preventive use of antibiotics and not to their curative use. In addition, this concern came mainly from the participants of the advisory group (P15b). Although participants recognized the use of preventive antibiotics on farms as excessive (P32a) they considered this an acceptable resource to deal with situations of stress within the herd (P1b).

#### Pork chain ambiguities in the AMU-AMR relationship and their impact on Brazilian consumers

Some of the participants showed skepticism regarding the link between AMU in animal production and AMR in humans. There were more expressions of concern about the damage caused by the withdrawal of AMU for prophylactic purposes than about possible risks to public health from AMU in the livestock sector. Some claimed that more scientific evidence is required to discuss the role of the livestock on the spread of AMR. Without this evidence, some argued, it would not be correct to impose restrictions on the use of veterinary antibiotics (P8a, P15c). However, others recognized the relationship between AMU in livestock and AMR and advocated for a stricter conduct in relation to the purchase and sale of veterinary antibiotics (P9a).

### Economic pillar—“It is cheaper to raise pigs using antibiotics”

Participants raised concerns regarding the loss of productivity in the absence of growth promoters, as well as the increase in production costs in the case of restrictions to AMU, and losses in income arising from the sale of antibiotics. Growth promoters were seen as essential, as if they were the miraculous solution for the production of cheap pork. Participants linked to agribusinesses and nutrition companies (health veterinarians, field technicians and nutritionists) stated that international markets were pressuring agribusinesses to abandon the use of antibiotic growth promoters (P30c). Many participants considered the withdrawal of antibiotic growth promoters as a lost battle, and some believed that the ban would open space for the development of new pharmaceutical additives, such as probiotics, to replace antibiotics (P1c). In general, participants predicted that a ban of AMU for growth promotion would reduce productivity on farms (P15d). Participants believed that the Brazilian market would not absorb an increase in the price of meat produced with reduced AMU (P6a). Likewise, low pork production cost was seen as a condition for competition in international markets (P8b).

Participants also said that to reduce AMU on the farms it would first be necessary to improve pigs' health and animal welfare. Yet, a key point addressed by stakeholders of all groups was that improving pig welfare to reduce current AMU requires investments, with no expectation of financial return (P12a). Improving farm infrastructure to reduce animal stress was considered costly and financially disadvantageous for farmers and agrobusiness (P9b). Antibiotics were described as part of the infrastructure needed to ensure animal welfare (P8c), which supported concerns expressed by some participants that reducing preventive AMU would be detrimental to the pigs' welfare (P14a). This was further justified by the concept of animal welfare expressed by the participating stakeholders, centered on thermal comfort, good productivity and avoiding disease (P6b, P5a). Only a few participants believed that high AMU was related to low levels of pig welfare on farms (P1d, P9c).

Finally, participants described employment and income relationships arising from the sale of antibiotics. Some participants reported a relationship of dependence of veterinarians, agronomists and animal scientists with pharmaceutical laboratories, either through jobs as commercial representatives or from the sale of veterinary medicines (P14b, P10a).

Brazilian consumers' lack of information or interest in AMR was cited to reinforce the narrative that the cost of changing AMU would not be viable (P23a, P17b). Some participants acknowledged that a segment of the public was concerned with AMU and therefore food safety in products of animal origin ought to be considered; however, they viewed this public as a specific niche market with greater purchasing power, which they considered less important and not representative of the Brazilian population (P18a). Others saw a trend for change in consumer behavior and a rise in new generations of consumers more informed, with habits and quality requirements different from the previous generations (P1e).

### Antibiotic dependence—Changes in AMU can collapse the pig production chain

Participants demonstrated predominantly negative attitudes toward policies to restrict AMU in pig farming in Brazil; 69% of them considered that these restrictions would have disastrous impacts on the pig production chain (P30d). Even the few who viewed the current AMU use in Brazil as excessive assumed losses and identified the same barriers (economic, sanitary and structural) in the implementation of these policies as the participants that were unfavorable to changes. Still, although skeptical about the effectiveness of a restrictive AMU scenario in Brazil, some participants believed that changes would inevitably happen (P17c). Of these, most believed that changes in AMU would be mandatory and would happen from pressure from foreign markets (P21a). As pork exports are important for Brazilian agribusiness, some of them assumed that legislation would meet international requirements and that agrobusiness would be forced to adapt (P5b).

### Changing the law is easier than enforcing it

The participants considered that the starting point to implement policies to restrict AMU in Brazil should be to control the purchase and sale of veterinary drugs (P28a). They also believed that the success of such measures depended on effective inspection of agricultural houses and other businesses by Brazilian health agencies (P1f, P31b). Further, participants pointed out that the success of restrictive measures of AMU depended on training veterinarians and farmers on good production practices and prudent AMU (P21b). Animal health defense professionals reported that governmental animal health agencies were already organizing discussions on the topic, with health education included in the strategies for planning for prudent AMU in Brazil. Some participants linked to the support group (researchers and animal sanitary defense professionals) believed that legislative changes could meet political resistance. They mentioned the role of parliamentarians related to pharmaceutical groups or agrobusiness and believed that political impasses could hamper the progress of technical proposals aiming at prudent AMU in the country (P9d).

## Discussion

Our study showed that antibiotics play a structural role in the Brazilian pig production chain, far beyond their pharmacological benefits. It also revealed an intricate network of stakeholders who depend on the social and financial relationships that antibiotics provide. These relationships underpin the lack of autonomy or motivation of these stakeholders to contribute to making changes in the use of veterinary antibiotics, identified in this research. The barriers raised by the participants to policies restricting AMU seem to reflect how much the pig production chain depends on these drugs for profitability and job opportunities. This fits with what Chandler (2) and Kirchhelle (37) described as a structure, sometimes invisible, based on antibiotics. The deeply rooted associations between the different social actors and antibiotics make it difficult for these stakeholders to perceive themselves as part of the problem.

Most of the stakeholders in our study believed that AMU in pig production could be considered excessive although there is no easily accessible evidence or official records of the use of veterinary antibiotics in Brazil to support this widely held view (38). However, some studies have reported the use of highly critical important antibiotics, broad spectrum antimicrobial for group prophylaxis and group/individual treatment of pigs, use of the agents in high dosages and for long periods in pig farms in Brazil (16, 35, 39).

Stakeholders expressed greater concerns with the economic and productive consequences of withdrawing antibiotics than with AMR risks and demonstrated low motivation to tackle the causes that may lead to the abuse of AMU, such as poor animal welfare and biosecurity status. We also identified a collective representation among the participants of antibiotics as a “magic” or “miraculous” solution to complex structural problems, similar to described by Chandler (2). Willis and Chandler (40), in their ethnographic work, defined antibiotics as a “quick fix for care in fractured health systems; a quick fix for productivity at local and global scales, for humans, animals and crops; a quick fix for hygiene in settings of minimized resources”. As our results show, the current AMU by the pig industry interlocks the economy of individuals and communities, political will, and social norms (16, 35). Therefore, the success of any public policies to respond to the AMU/AMR problem demands input from social research that explores the engines of AMR and also for the critical players to act up on this evidence.

Although participants acknowledged the environmental spread of AMR caused by AMU in pig production, most did not believe that there is a clear link between pig AMU and the public health concern with AMR (41). Other authors have demonstrated this type of belief “or more so a disbelief” among veterinary professionals (42, 43). By stating that there is a lack of evidence regarding livestock's role in the spread of resistant bacteria, professionals in the pig industry maintain the status quo of their practices and defend their “territory” (44). This perception of the livestock sector's low (or lack of) responsibility transfers the problem to other spheres, in this case, human medicine. Such fragmentation of the AMR problem makes it difficult to attribute ownership of the problem, which generates controversies in establishing cause and effect relationships, in the elaboration of regulations, and in the execution of contingency plans (3, 44).

The veterinary health professionals discussed AMU to prevent disease as an acceptable practice, a view shared by pig farmers in the same (16) and in other communities (45, 46). Similar views have been reported among medical doctors and veterinarians from low- and middle-income countries (Ethiopia, India, Nigeria, the Philippines, Sierra Leone and Vietnam), where health professionals, although aware of the risks of AMR, preferred to prescribe broad-spectrum antibiotics than to adopt measures that facilitate rational AMU (34). These factors may be related to cultural and geographical problems, as in some regions the capacity to ensure good hygiene, biosecurity and good animal welfare is limited (6). Professionals as those in the operational and advisory positions in this study are the main source of reliable information for farmers. Therefore, they must be the first advocates of prudent AMU measures. Establishing an effective dialogue between these actors and farmers is essential, given that they play a role as veterinary authorities vis-à-vis farmers (47). The complexity of the relationships amongst social actors, their different functions and interests, added to the space, materials and time difficulties between the decision-making and the results in the field, magnify discrepancies and lack of motivation of farmers to collaborate (3). Therefore, this dialogue must follow a proactive approach, with a unified, unambiguous message, consistent with the reality of the farmers (47, 48).

Believing that Brazilian consumers were not sufficiently informed or concerned with AMR and AMU, the participants showed disinformation and disconnection from the public. This belief is shared by pig farmers (35) and stakeholders of the dairy chain (49) in Brazil. However, some studies have shown that Brazilian citizens are increasingly interested and informed about production systems, animal welfare, and especially with chemical inputs used in food production, including antibiotics (50, 51). Based on the belief that consumers are uninformed, many participants resisted establishing an honest dialogue with this public, including many of the stakeholders that represented important institutions in the supply and inspection of food, and therefore are in the position to do so.

Many practices and attitudes reported in the present study were identified in the companion studies with farmers, both concerning AMU and AMR (16) and pig welfare (35). These include the conception of pig welfare restricted to biological functioning and productivity, the inability to associate animal welfare with the AMU problem, the reliability on AMU as a preventive health tool, and the description of antibiotics as part of the material infrastructure that supports cheap pork production. Also, several problems related to animal welfare and biosecurity in pig farms and easy access to antibiotics, mentioned by stakeholders in the present study as a reason for excessive AMU, are confirmed in these and other studies in the region (52, 53). Just as farmers (16) this study participants did not trust the effectiveness of policies to restrict AMU in Brazil, assuming a collective lack of willingness to comply. The conflict of interest of veterinarians that make a profit from prescribing antibiotics, also reported by farmers as a reason for loss of trust in these stakeholders (16), undermines their potential to contribute to change AMU practices. Altogether, mutual blame shifting among the stakeholders of the production chain exposes the lack of motivation to assume responsibility to change, whereas common views may act to legitimize behaviors that sustain current AMU. This in turn may explain the collective view that the most effective force of change in AMU may come from external markets.

Specific actions that cause individual behavioral changes in AMU can help create an idea of collective awareness about the problem of bacterial resistance (3). However, the reckless AMU and control of AMR should not be seen only as a problem of individual behavior of these professionals or the farmers. Altogether, our findings indicate the need for collective and transformative actions to tackle the AMU/AMR problem, as discussed by Chandler (2). It will be up to Brazilian and international health agencies to engage more comprehensively, based on interdisciplinary and systemic actions that should go beyond health aspects (1, 54). The One Health initiative, which is part of the Global Action Plan to Combat AMR (28) is an example that encourages the development of systemic strategies between human, animal and environmental health (54).

## Conclusions

The views of high-level professionals in the Brazilian livestock chain reveal antibiotics as a structural element that enables pig production. Stakeholders rationalized antibiotics as essential to maintain production by preventing and treating diseases that derive from uncontested stressful production practices driven by the industry's goals of production quantity over quality. Production and related financial returns create a web of complicity and a rooted unwillingness to break or change the status quo, underpinned by fear of losing economic gains or social license among peers. As individuals, stakeholders sensed a need to reduce/change AMU practices; nevertheless, this was mainly driven by what they saw as international pressure, rather than genuine public health concerns. Stakeholders accepted risky AMU practices, shifting the blame to farmers, politicians, and consumers, which indicates that successful measures to deal with the AMU/AMR challenges in the pig chain shall not be rooted in personal behavior change. Instead, honest interdisciplinary dialogues and structural changes are needed to define common grounds and a way forward to break the cycle that perpetuates antibiotics as structural commodities. We believe that this study and similar future studies lay a baseline for starting such dialogues. Further, we discourage the idea (popular among the study participants) that external markets could become a positive driving force for change. We foresee that such a top-down approach is, in fact, a threat to the sustainability of changes within the industry, as it may disrupt the livelihoods of small/family farmers, disregard concerns of local markets (i.e., Brazilian consumers), and not necessarily improve the welfare of pigs, the often-voiceless stakeholder with an intrinsic value we ought to advocate for in these conversations.

## Data availability statement

The datasets presented in this article are not readily available because not applicable. Requests to access the datasets should be directed to rita.silva@ifc.edu.br.

## Ethics statement

The studies involving human participants were reviewed and approved by Comitê de Ética em Pesquisa com Seres Humanos (CEPSH) - UFSC. The patients/participants provided their written informed consent to participate in this study.

## Author contributions

RA-G and GOA: conceptualization, data curation, investigation, methodology, validation, visualization, writing—original draft, and writing—review and editing. MH: conceptualization, data curation, funding acquisition, investigation, methodology, project administration, resources, supervision, validation, visualization, writing—original draft, and writing—review and editing. All authors contributed to the article and approved the submitted version.

## Funding

This study was supported by CNPq (National Council for Scientific and Technological Development, Brazil, Grant No. 404403/2016-6). MH was supported by CNPq through Grant No. 304968/ 2019-6.

## Conflict of interest

The authors declare that the research was conducted in the absence of any commercial or financial relationships that could be construed as a potential conflict of interest.

## Publisher's note

All claims expressed in this article are solely those of the authors and do not necessarily represent those of their affiliated organizations, or those of the publisher, the editors and the reviewers. Any product that may be evaluated in this article, or claim that may be made by its manufacturer, is not guaranteed or endorsed by the publisher.
