# Supplementary material for: Waiting for markets to change me—High-stakeholders' views of antibiotic use and antibiotic resistance in pig production in Brazil
Source: Front Vet Sci. 2022 Sep 16;9:980546. doi: 10.3389/fvets.2022.980546 (PMC9523568; doi:10.3389/fvets.2022.980546)
Supplement: Supplementary file 1 [file Table_1.DOCX]

*Supplementary Material*

Waiting for markets to change me – High-stakeholders’ views of antibiotic use and antibiotic resistance in pig production in Brazil

Rita Albernaz-Gonçalves^1,2^, Gabriela Olmos Antillón^3^, Maria José Hötzel^1^

*** Correspondence:** Maria José Hötzel [maria.j.hotzel@ufsc.br](mailto:maria.j.hotzel@ufsc.br)

**Script interview model (Areas to be explored with interview)**

1. How important are antibiotics for animal production?
2. What do you consider to be the rational use of antibiotics?
3. Does Brazilian pig farming use antibiotics rationally?
4. Definition of bacterial resistance to antibiotics?
5. Relationship between AMU in animals and AMR in humans
6. Do consumers worry about AMU in animals?
7. Knowledge of prudent AMU policies
8. Definition of animal welfare
9. Is animal welfare related to AMU?
10. Assuming a hypothetical scenario in which Brazil would adopt measures of prudent AMU-like international models (prohibition of antibiotics to promote growth and restriction of prophylactics).
    1. What would this scenario look like in Brazilian animal production?
    2. What is the feasibility of this scenario?
    3. What measures would be necessary to adopt these measures?
